# Miniature Fabry-Perot Cavity Based on Fiber Bragg Gratings Fabricated by Fs Laser Micromachining Technique

**DOI:** 10.3390/nano11102505

**Published:** 2021-09-26

**Authors:** Luohao Lei, Hongye Li, Jing Shi, Qihao Hu, Xiaofan Zhao, Baiyi Wu, Meng Wang, Zefeng Wang

**Affiliations:** 1 College of Advanced Interdisciplinary Studies, National University of Defense Technology, Changsha 410073, China; leiluohao18@nudt.edu.cn (L.L.); shijing18@nudt.edu.cn (J.S.); huqihao18@nudt.edu.cn (Q.H.); zhaoxiaofan_zxf@nudt.edu.cn (X.Z.); wubaiyi@nudt.edu.cn (B.W.); wangmeng@nudt.edu.cn (M.W.); 2 State Key Laboratory of Pulsed Power Laser Technology, National University of Defense Technology, Changsha 410073, China; 3 Hunan Provincial Key Laboratory of High Energy Laser Technology, National University of Defense Technology, Changsha 410073, China

**Keywords:** Fiber Bragg gratings, Fabry-Perot cavity, femtosecond laser, micromachining technique

## Abstract

Fabry-Perot cavity (FPC) based on Fiber Bragg gratings (FBGs) is an excellent candidate for fiber sensing and high-precision measurement. The advancement of the femtosecond laser micromachining technique provides more choices for the fabrication of FBGs-based FPCs. In this paper, we fabricate miniature FBGs-based FPCs, using the femtosecond laser line-by-line scanning writing technique for the first time. By this method, the FBGs can be limited to a specific area in the fiber core region. The grating length, position, and the distance between two successive FBGs can be conveniently controlled to achieve the desired transmission spectrum. For future applications in sensing, the temperature and strain responses of the fabricated FBGs-based FPCs were studied experimentally. This work provides a meaningful guidance for the fabrication and application of miniature FPCs based on FBGs.

## 1. Introduction

Fiber-optics Fabry-Perot cavity (FPC) is an important kind of optical device and is widely applied for optical sensing, high precision measurement, and digital signal processing, due to the characteristics of compactness, high sensitivity and immune to electromagnetic interference [1]. To date, many structures of FPCs have been demonstrated, including two inserted vertically cut fibers [2], hybrid structure based on large lateral offset splicing and cleaving [3], two FBGs with the same resonant wavelength [4], etc. Among them, FBGs-based FPCs are relatively more compact and stable as well as being easier to control the cavity parameters in fabrication. So, this kind of FPC plays an important role in fiber sensing. For example, they can be used as pickup for acoustic guitars [5]; a magnetic field detector could be realized by recording the wavelength shift [6]; and they can also be applied as a high-resolution inclinometer [7].

The fabrication of FBGs-based FPCs can be realized by cascading or partly superimposing multiple FBGs on fibers [8]. Ultraviolet exposure is the most commonly used method to inscribe FBGs with which FBGs often present high stability and low insertion loss. However, the resonant wavelength is decided by the period of the used phase mask, and hydrogen-loading and annealing are unavoidable processes. Direct ultraviolet writing was reported to fabricate miniature FPCs in waveguides [9,10], but for fiber-based inscription systems, the fabrication of the microcavity (total length of FPC in the magnitude of millimeters) is extremely difficult; for example, the total length of cavity in [8] was larger than 10 cm. Owing to the development of the femtosecond laser direct writing technique [11,12,13,14], hydrogen loading and annealing are not necessary anymore, and the resonant wavelength is not restricted by the phase mask. Until now, FBGs-based FPCs by femtosecond laser direct writing were reported in few articles. For instance, femtosecond laser point-by-point inscription was employed to manufacture FBGs-based FPCs [15], which is the most convenient and timesaving method. However, due to the influence of transmission loss caused by the Mie scattering effect [16], the insertion loss and the polarization-dependent loss in this method, a part of the core guided energy coupled to the cladding layer, the spectrum was not symmetric, and the interference was not obvious. Femtosecond laser plane-by-plane inscription [17,18,19] was also reported for the fabrication of FBGs-based FPCs in multimode polymer fiber [20] in which the spectrum of FBGs-based FPCs is relatively smooth, but the inscription process is relatively time-consuming. Femtosecond laser line-by-line inscription is another efficient method to fabricate FBGs [21,22,23]. However, up to now, no FBGs-based FPCs fabricated by this method were reported. Compared with point-by-point and plane-by-plane inscription methods, line-by-line writing can achieve a balance between a good spectrum, time consumption and the complexity of the writing system.

In this paper, we theoretically study the spectral properties of FBGs-based FPCs, and simulations are performed to explore how three important fabrication parameters of the cavity length, grating length and refractive index modulation affect the transmission spectra. Based on the simulation, four miniature FBGs-based FPCs are fabricated by the femtosecond laser line-by-line inscription method for the first time, and the total length of FPCs is in the magnitude of millimeters. To investigate the properties of the fabricated FPCs in the future sensing applications, two systems are constructed for testing the temperature and strain responses of the FPCs. The experimental results show that the miniature FBGs-based FPCs fabricated here have very stable characteristics, which is helpful for future applications. Our research provides a new method for the fabrication of miniature FBGs-based FPCs and facilitates the fabrication of highly integrated optical fiber devices in the future.

## 2. Theories and Simulations

**Figure 1:**
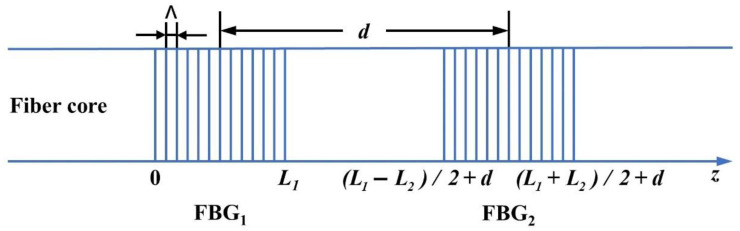
Schematic of a FBGs-based FPC.

The schematic of a FBGs-based FPC is shown in Figure 1. Two FBGs constitute the structure. Λ is the period of FBGs, *L* is the grating length, *d* is the cavity length (the distance between the center of FBG_1_ and FBG_2_) and the total length of FBGs-based FPC is *d* + (*L*_1_ + *L*_2_)/2.

The transmission coefficient *U_t_* and transmission rate *I_t_* of the FBGs-based FPC can be given by the following:

(1) $$U_{t}=\frac{t_{1}t_{2}}{1-r_{1}r_{2}e^{i\delta }}$$

(2) $$I_{t}=U_{t}U_{t}^{*}$$

where, *r*_1_ and *r*_2_ are the reflection coefficients of FBG_1_ and FBG_2_; *t*_1_ and *t*_2_ are the transmission coefficients of FBG_1_ and FBG_2_; *δ* is optical path difference, *δ* = 4*πn_eff_* [*d* − (*L_1_* + *L_2_*)/2]/*λ,* where *n_eff_* is the effective refractive index of the fiber grating, *λ* is the wavelength of the incident light, * represents conjugate operations.

**Figure 2:**
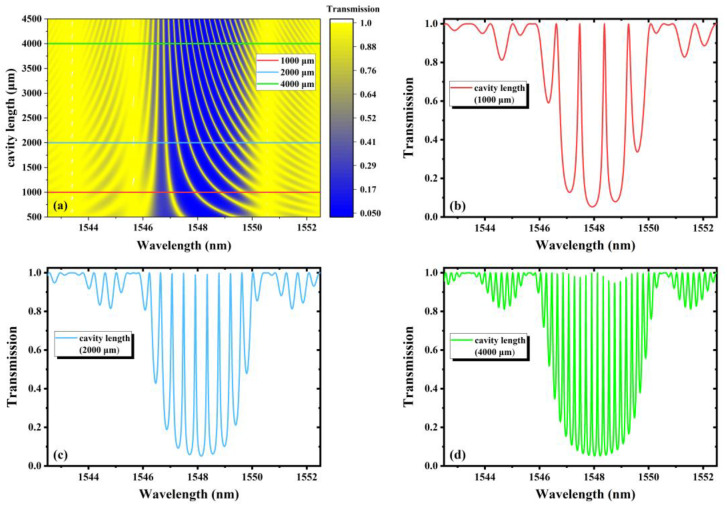
(**a**) The transmission spectra with continuous variation in cavity length from 500 μm to 4500 μm; the transmission spectra with cavity length of (**b**) 1000 μm, (**c**) 2000 μm and (**d**) 4000 μm.

**Figure 3:**
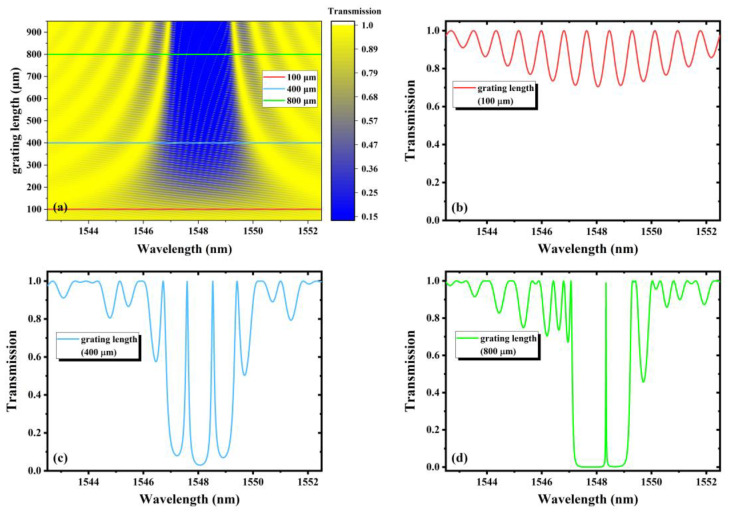
(**a**) The transmission spectra with continuous variation of grating length from 50 μm to 950 μm; the transmission spectra with grating length of (**b**) 100 μm, (**c**) 400 μm and (**d**) 800 μm.

**Figure 4:**
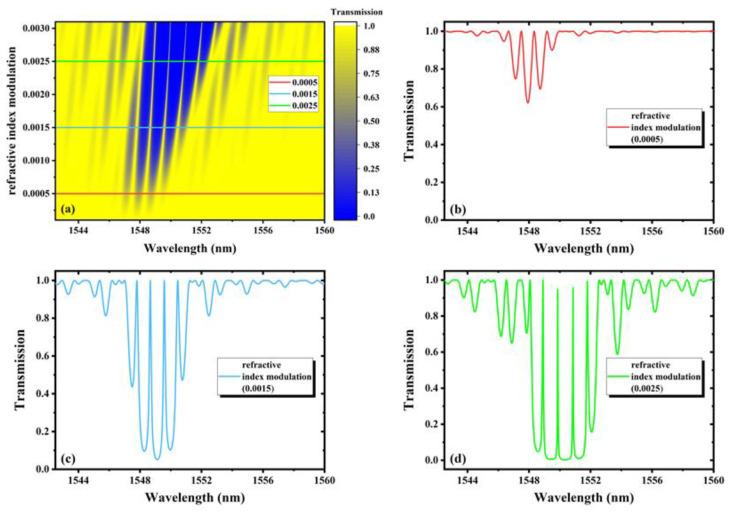
(**a**)The transmission spectra with continuous variation of the refractive index modulation from 0.0001 to 0.003; the transmission spectra with the refractive index modulation of (**b**) 0.0005, (**c**) 0.0015 and (**d**) 0.0025.

From Equations (1) and (2), the evolutions of the transmission characteristics of FBGs-based FPCs with the parameters can be simulated. In the following research, according to the actual writing parameters, the cavity length, grating length and refractive index modulation are selected for simulation, exploring how these parameters’ change affects the transmission spectra, as shown in Figure 2, Figure 3 and Figure 4 respectively.

Figure 2a shows the transmission spectra of FBGs-based FPCs with continuous variation in the cavity length from 500 μm to 4500 μm. The grating length is fixed at 355.24 μm, and the refractive index modulation is 0.0015. The interference not only occurs in the main interference band, but also in the sidelobes. It is seen that as the cavity length increases, the number of rejection bands in the main interference band increases, and the width of each rejection band becomes narrower. Figure 2b–d shows the transmission spectra under three different cavity lengths of 1000 μm, 2000 μm and 4000 μm, respectively. Here, we define each maximum transmittance as a peak, and each minimum transmittance as a dip. We can see that the number of peaks and dips increase with the increasing of the cavity length. However, the envelope of the spectra does not change because the envelope only depends on the FBGs comprised of the FPCs, and in our simulation, the grating length and the refractive index modulation remain unchanged. We can also see that the cavity length becomes longer and the free spectral range (FSR) becomes narrower.

The transmission spectra of continuous variation grating length from 50 μm to 950 μm are shown in Figure 3a. The distance between the center of two FBGs is fixed at 1000 μm, and the refractive index modulation is 0.0015. Figure 3b–d shows the transmission spectra when the grating length are 100 μm, 400 μm and 800 μm, respectively. It can be seen that as the grating length increases, the width of the main interference band and sidelobe becomes narrower, while the bandwidth of the peak closest to the central wavelength of 1548 nm also becomes narrower and narrower. For a single grating, a shorter grating length decides a weaker reflectivity and a wider bandwidth [24], giving weaker dips as shown in Figure 3b. If the grating length becomes longer, the symmetry of the transmission spectrum degrades, as shown in Figure 3d. The reason is that the distance between the FBGs decreases when the grating length increases, as the cavity length is fixed in simulation, which contributes to the decrease in the optical path difference *δ*, causing detuning of the cavity. Thus, a proper grating length is needed for sensing.

Figure 4a represents the transmission spectra under the condition of continuously changing refractive index modulation from 0.0001 to 0.003. Figure 4b–d corresponds to the transmission spectra under the condition of modulating the refractive index of 0.0005, 0.0015 and 0.0025, respectively. The grating length is 355.24 μm and the cavity length is 1000 μm. With the increase in refractive index modulation, the central wavelength shifts red and the resonant intensity becomes stronger rapidly. Additionally, the width of the main interference band and sidelobe becomes wider, and the peaks become narrower. However, the number of peaks and dips do not change, although the FSR becomes wider. Therefore, a FBGs-based FPC with deeper dips and narrower peaks can be fabricated by increasing the refractive index modulation, which is realized by adding the femtosecond laser pulse energy, repetition rate, or writing time. However, the insertion loss is an important parameter that needs to be considered.

In summary, the bandwidth of each peak and the FSR becomes narrower with the increase in the cavity length or grating length. However, both of them become wider, while the refractive index modulation increases. The width of the main interference band and sidelobes is only decided by the grating length and the refractive index modulation. The depth of the dips becomes deeper by increasing not the grating length, but the refractive index modulation. In the following part, we fabricate FBGs-based FPCs, using femtosecond line-by-line inscription. The simulation results could provide some useful guidance for fabrication.

## 3. Fabrication

**Figure 5:**
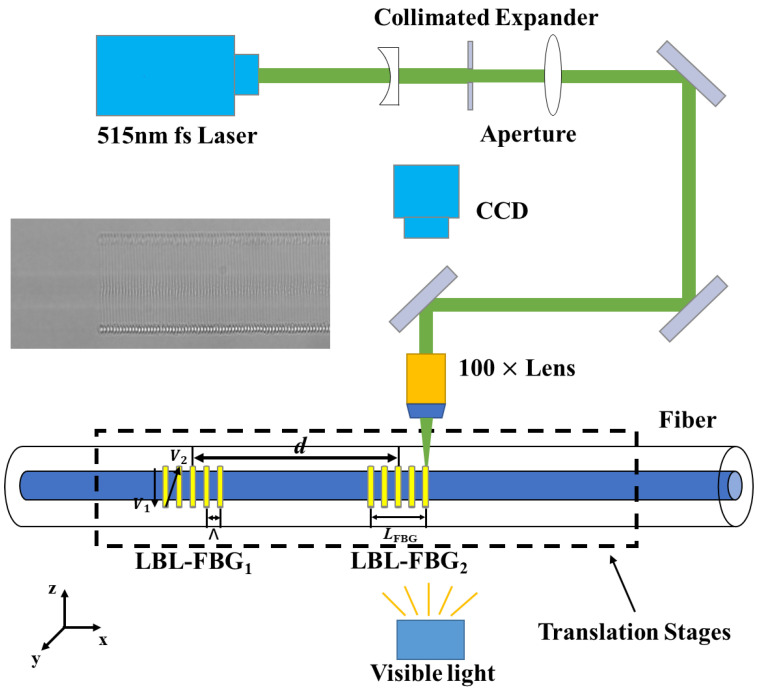
Schematic of the fs laser line-by-line scanning writing system. Inset: microscopic image (100×) of single FBG.

Figure 5 represents the schematics of the fs laser line-by-line writing system, composed of an fs laser, light control system, oil-immersion lens, high-precision fiber control platform, and fiber core imaging system. The fs laser operates at 515 nm with a repetition rate of 1 kHz, which is frequency doubled from a 1030 nm solid laser. After passing through the light control system composed of a collimator, an aperture, an expander, and three mirrors, the fs laser is finally focused into the fiber core by the oil-immersion lens (magnification of 100×). As fs writing requires very high stability and accuracy, the fiber is fixed on a high-precision 3D electrical translation stage. Meanwhile, the fiber core imaging system is used to observe where the laser is focused during the inscription. The resolution of each line is 0.7 μm. The scanning speed is 1 mm/s, and the diameter of the elementary point is about 0.7 μm (the diameter of the focal spot for the “oil-immersion lens” is about 1μm). The refractive index modulation generated by femtosecond laser is in the magnitude of 10^−3^. Fluctuations in the transverse dimension of the grating do exist but exert little influence on the spectrum of FPCs. The microscopic image (100×) of a single FBG is illustrated in the inset of Figure 5.

The schematic diagram of the writing parameters is shown in Figure 5, where the grating length is *L*_FBG_ and the distance between the centers of the two gratings is *d*. The period of the FBG is set as 1070 nm, which can realize the second order resonance at the wavelength of ~1550 nm. The length of each line is about 32 μm. By varying the line position over the cross section of the fiber core, the coupling coefficient of the fundamental mode will change in the meantime. If the line is located in the center of the core, the coupling coefficient will be strong. Off-center inscription will decrease the coupling coefficient. In the inscription, the pulse energy is about 36 nJ. After finishing the inscription of LBL-FBG_1_, the 3D electrical translation stage translates along the fiber axial direction, and the displacement distance is *d*—*L*_FBG_. Then, LBL-FBG_2_ begins to be inscribed. The length of LBL-FBG_2_ is equal to that of LBL-FBG_1_, and both are located in the same plane, parallel to the fiber axis. The two FBGs have the same writing time length. Thus, the spectra of these two LBL-FBGs are almost identical. To control the insertion loss and for comparison, the transmission depth near the central wavelength of each FBG is fixed to be about 1 dB.

**Figure 6:**
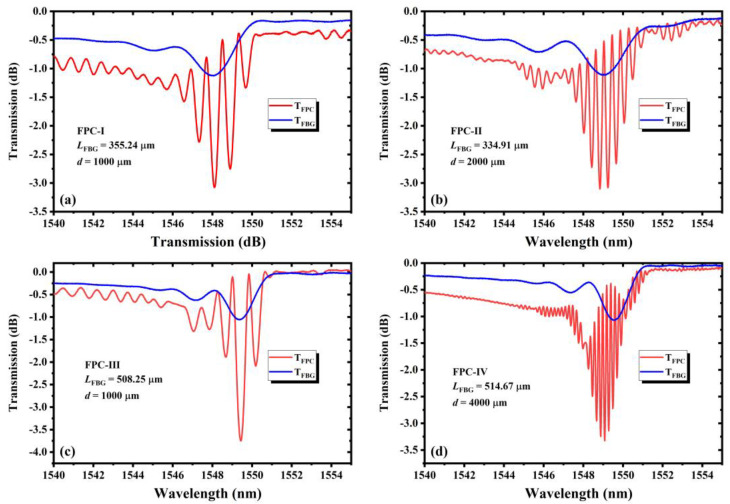
The transmission spectra of four line-by-line inscribed FBGs-based FPCs. (**a**) FPC-I: grating length 355.24 μm, cavity length 1000 μm; (**b**) FPC-II: grating length 334.91 μm, cavity length 2000 μm; (**c**) FPC-III: grating length 508.25 μm, cavity length 1000 μm; (**d**) FPC-IV: grating length 514.67 μm, cavity length 4000 μm.

Figure 6 shows the spectra of four different FBGs-based FPCs. Figure 6a shows that the FSR is about 0.8 nm and there are five dips within the Bragg resonance wavelength range. Figure 6b illustrates that the FSR downsizes to 0.4 nm and the number of dips increases to 10. Comparing Figure 6a,b, the length of LBL-FBG is close, which indicates that the coupling coefficient is also close, as we fix the same refractive index modulation intensity (showing by the same transmission depth of about 1 dB). Under this condition, if the cavity length increases, the FSR decreases. When the coupling coefficient decreases (even if the grating length increases in this condition), the FSR also decreases. As shown in Figure 6c, it can be derived from the spectra that the FSR is about 0.75 nm. The experimental tendency agrees well with the theoretical tendency. A narrower FSR can be derived by decreasing the coupling coefficient or increasing the cavity length. For instance, as illustrated in Figure 6d, the FSR is only 0.2 nm. The bandwidth of the peak also conforms to the same law. In summary, the experimental results are well consistent with the simulation results. The occurrence of additional dips in transmission spectrum has something to do with the sidelobe of FBGs. As we can see in Figure 2 in the manuscript, interference also appears out of the Bragg resonance range, especially the sidelobe range. For the reason that FBGs can also reflect light in the sidelobe range, this provides the condition for the appearance of FP interference if two identical FBGs are inscribed on the same fiber. Moreover, strong cladding mode coupling occurs in the line-by-line inscribed FBG, the dips in the cladding mode coupling region are also influenced by the transmission loss, and the whole spectrum is not symmetric.

## 4. Temperature and Strain Response Measurement

To investigate the properties of the fabricated FPCs in future sensing applications, two systems are constructed for testing the temperature and strain responses of the FPCs. In the following research, FPC-I and FPC-IV in Figure 6 are selected for the experiments to compare the characteristics of FPCs of different FSRs, which could provide some useful guidance for future temperature or strain sensing.

### 4.1. Temperature Response

**Figure 7:**
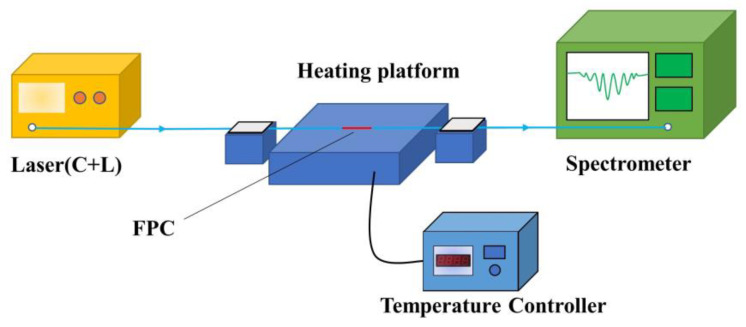
The schematic configuration of the temperature response test system.

The temperature response test system is shown in Figure 7. The laser covering the C- and L-band passes through the FPC from the light source (VASS-CL-B, Connect Ltd. Shanghai, China) to the spectrometer (AQ6730D, Yokogawa Ltd. Tokyo, Japan). At the same time, the FPC is clamped in the center of the heating platform, which is an intelligent temperature control platform (P-3020/P-3020A, AntaiX Ltd. Shenzhen, China), with an accuracy of 0.1 °C to ensure uniform heating. Finally, the spectrum is recorded from low temperature to high temperature continuously.

**Figure 8:**
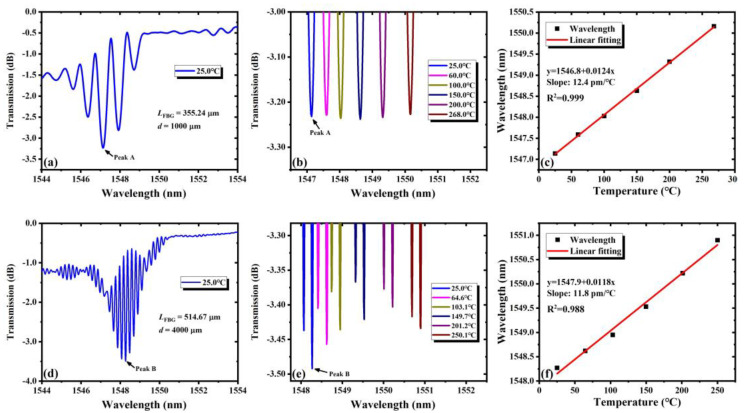
Results of the measured temperature response characteristics: (**a**–**c**) FPC-I; (**d**–**f**) FPC-IV. (**a**,**d**) The transmission spectra; (**b**,**e**) the resonance peaks shift with temperature; (**c**,**f**) the linear fitting of the peak wavelength shift.

The results of the temperature response are shown in Figure 8. Figure 8a,d shows the measured transmission spectra of FPC-I and FPC-IV at room temperature, respectively. Peaks A and B represent the minimum of the transmission of the two FPCs respectively. Figure 8b,e shows the wavelength shift of Peaks A and B with six different temperatures. It is found that the peaks have a red shift when the temperature increases, and Figure 8c,f shows the fitting curves, showing a good linear relationship between the wavelength shift and the variation in temperature. The sensitivity of the wavelength drift is 12.4 pm/°C for PCF-I and 11.8 pm/°C for PCF-IV. In view of that, the resolution of the parameter measurement depends on the minimum resolution of the spectrometer, so the resolution of the temperature resolutions of both PCFs are 0.81 °C and 0.85 °C, respectively. In actual applications for highly sensitive temperature measurement, the FPCs of narrower peaks and narrower FSR will have higher sensitivity. Experimental results show that the FPCs work stably, even when the temperature exceeds 250 °C. The temperature is limited by the heating platform in experiments. In fact, we believe that the FPCs could work stably at a much higher temperature, which has important special applications in high-temperature measurement. This is one of the main advantages of FBGs fabricated by the fs laser writing technique, compared with the ultraviolet exposure method.

### 4.2. Strain Response

**Figure 9:**
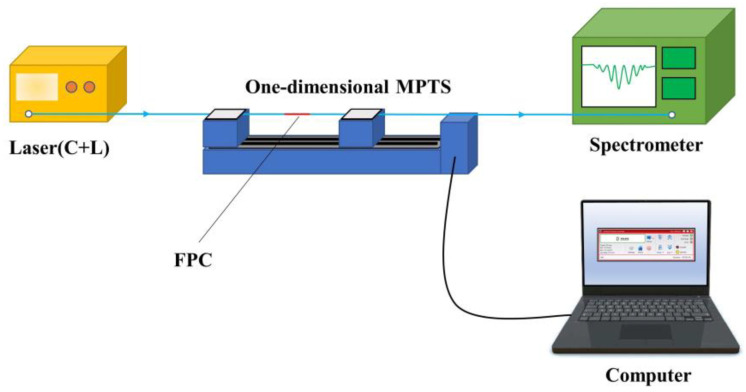
The schematic configuration of the strain response test system.

The strain response test system is shown in Figure 9. The FPC is fixed on a one-dimensional nanometer motorized high-precision translation stage by an optical fiber clamp. The computer controls one end to move a fixed distance in the same direction each time. In this way, the FPC is moved for a fixed distance to be elongated, and the spectrum is recorded at this time.

**Figure 10:**
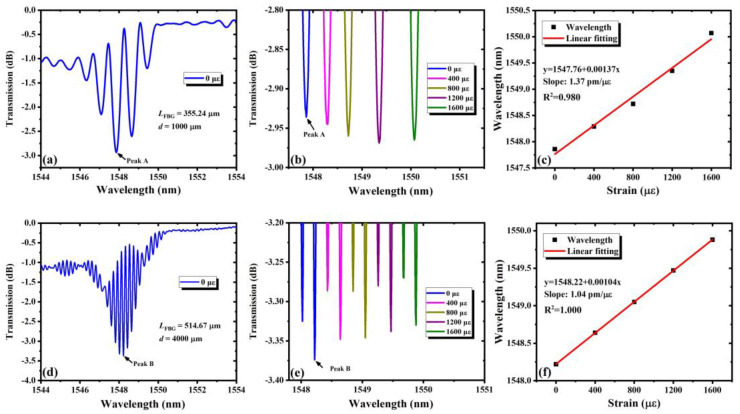
Results of the measured strain response characteristics: (**a**–**c**) FPC-I; (**d**–**f**) FPC-IV. (**a**,**d**) show the transmission spectra; (**b**,**e**) show the resonance peaks shift with strain; (**c**,**f**) show the linear fitting of the peak wavelength shift.

The results of the strain response are shown in Figure 10. In principle, the external strain loaded on the FPC will change the cavity length and the grating length, which will influence the transmission spectrum of the FPC. Figure 10a,d shows the measured transmission spectra of FPC-I and FPC-IV when no external strain is exerted, respectively. Peaks A and B represent the deepest peak in the two transmission spectra respectively. Figure 10b,e shows the wavelength shift of Peaks A and B as the loaded strain increases. It is found that the peaks have a red shift when the strain increases, just like the response to the temperature, and Figure 10c,f shows the fitting curves, also showing a good linear relationship between the wavelength shift and the loaded strain. The sensitivity of the wavelength drift is 1.37 pm/με for PCF-I and 1.04 pm/με for PCF-IV. In a similar way, the strain resolutions of both PCF are 7.3 με and 9.6 με, respectively. Results show that FPCs of wider FSR, usually having shorter cavity lengths, would have higher sensitivity to strain. This may be due to the bigger relative change in the cavity and FBG lengths to the strain when the cavity is shorter. Experimental results also show that the FPCs work stably when the external strain changes.

## 5. Conclusions

Miniature FBGs-based FPCs fabricated by femtosecond laser line-by-line inscription method are reported for the first time. The transmission spectra characteristics of the FPCs are studied by simulations and experiments. The results agree well with each other and show that the bandwidth of each peak in the main interference and the free spectral range of the FPC become narrower with the increase in the cavity length or grating length. In addition, the temperature and strain response characteristics of the fabricated FPCs are also studied experimentally. Results show that the FPCs work stably, and the peak wavelength changes linearly with temperature or strain changes, which is of great significance to optical sensing in the future. Our studies come up with a new method to fabricate miniature FBGs-based FPCs, which is meaningful in new concept sensors and integrated optics.

## Data Availability

The data is available on reasonable request from the corresponding author.
